# Study on the Damage Mechanism of an H62-Cu/7075-Al Tribo-Pair Under the Influences of Current Direction and Density

**DOI:** 10.3390/ma17225395

**Published:** 2024-11-05

**Authors:** Pengfei Chen, Yanyan Zhang, Chenfei Song

**Affiliations:** National United Engineering Laboratory for Advanced Bearing Tribology, Henan University of Science and Technology, Luoyang 471023, China; pengfeicpf2000@outlook.com (P.C.); cfsong@haust.edu.cn (C.S.)

**Keywords:** copper/aluminum friction pair, arc ablation, frictional wear, oxidation, electromigration

## Abstract

In the present study, we used 7075 Al-H62 Cu and H62 Cu-7075 Al pairs to study the effects of current density and direction on their tribological properties and on the damage caused by the current-carrying friction and wear. We found that, when the current density increased from 0 A/mm^2^ to 79.61 A/mm^2^, the coefficients of friction for both pairs decreased. Results obtained after wear indicate that the current direction influences the electromigration between the two tracks, leading to different kinds of damage on the worn surface. In the case of the 7075 Al-H62 Cu pair, damage mainly involved mechanical wear at low current densities. As the current density increased, electro-erosion damage gradually became more dominant. Under the action of a large electric arc, the material surface was severely eroded, and a dense oxide film formed on the material contact surface, ultimately leading to the failure of electrical conduction between the materials. In the case of the H62 Cu-7075 Al pair, damage mainly involved mechanical wear. A layer of copper film was found on the surface of the worn aluminum pin, which caused its mass to be greater than it was before wear.

## 1. Introduction

**Figure 1:**
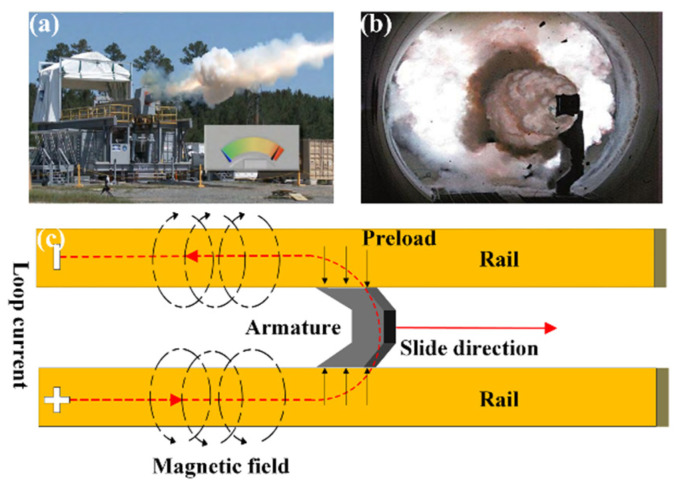
(**a**,**b**) Applications of railguns; (**c**) schematic of the railgun principle.

Electromagnetic launching is capable of overcoming the limitations of energy and speed associated with traditional launching methods. This new launching method has unique advantages, including greater launching kinetic energy, higher system efficiency, reduced start-up times, stronger sustained launching capability, and load adjustability [1,2]. During the operational process, the current flows through the armature from one track to the other, as shown in Figure 1. The loop current produces an induced magnetic field between two parallel tracks. The Lorentz force is generated, causing the armature to be launched at an ultrafast speed [3,4,5]. When the two tracks are subjected to higher current densities, larger electromagnetic loads, or greater mechanical and thermal impacts, damage is caused to the two tracks, including fracture, grooving, and arc erosion [6,7,8]. As a result, the service life of the tracks is shortened, reducing the fighting efficiency and increasing costs. These are the technical bottlenecks that have impacted the development of electromagnetic rail guns (EMRGs) [9].

To date, researchers have carried out numerous experiments on various aspects of EMRGs, including structural design, material development, and dynamic simulation. Xia Ge et al. established a numerical calculation model of armature rail contact voltage and contact resistance in an enhanced electromagnetic rail gun. Their model revealed a correlation between armature rail contact resistance and physical parameters, such as muzzle voltage, muzzle-induced voltage, rail current, rail mutual inductance gradient, armature speed, and armature displacement [10]. Shuo Ma et al. established a two-dimensional geometric model of an electromagnetic railgun and a three-dimensional geometric model of an electromagnetic railgun in a single-firing mode, and obtained the thermal, spatial, and temporal distribution characteristics for a cross-section of the rail armature contact region [11]. Zengji Wang et al. carried out further simulations with the 3D model and showed that the electromagnetic force induced by the negative net current in the tail of the armature was not the main cause of the transition [12]. At present, scholars mostly use numerical simulations to study electromagnetic guns. However, there is some research on the damage caused to copper–aluminum pairs by current-carrying friction. Jyh-Chia and Vook tested aluminum wire brushes on rotating copper slip rings and copper wire brushes on aluminum slip rings. The rings were rotated at 250 rpm for 10,000 revolutions at a maximum current of 30 A under high vacuum conditions. They found that the contact resistance was almost ten-times higher for an aluminum brush sliding on a copper slip ring compared with a copper brush sliding on an aluminum slip ring [13]. Dinesh G et al. studied the current-carrying friction of copper flat plates (C-110) and translational aluminum (Al-6061) pins with hemispherical and flat tips. Their results showed that the drop in voltage across the contact surface during sliding was negatively correlated with the friction. Measurements of wear pads on the pins showed that the wear diameters increased as the contact current increased [14].

The problem of multi-physics field coupling in EMRGs is not yet clearly understood. The mechanisms underlying the evolution of the physical parameters of materials also remain uncertain. It is therefore necessary to carry out research on the current-carrying friction of armature rail materials.

To investigate the impact of polarity changes on the friction and wear of the armature sides during the launching process, we used 7075 Al-H62 Cu and H62 Cu-7075 Al. At the same time, we varied the current magnitudes to study the influence of current on the structural evolution at the friction interface. The results show the influence of the current on friction and wear behavior, and provide a reference for further studies of EMRGs.

## 2. Materials and Methods

### 2.1. Test Equipment and Materials

The test rig used in this study was an FTM-CF100 tribometer manufactured by Henan University of Science and Technology (Luoyang, China) and Nanjing Bio-inspired Intelligent Technology Co., Ltd. (Nanjing, China) [15,16]. This tribometer was composed of a loading system, a high-speed drive system, a power supply system, and a measurement/control system.

**Figure 2:**
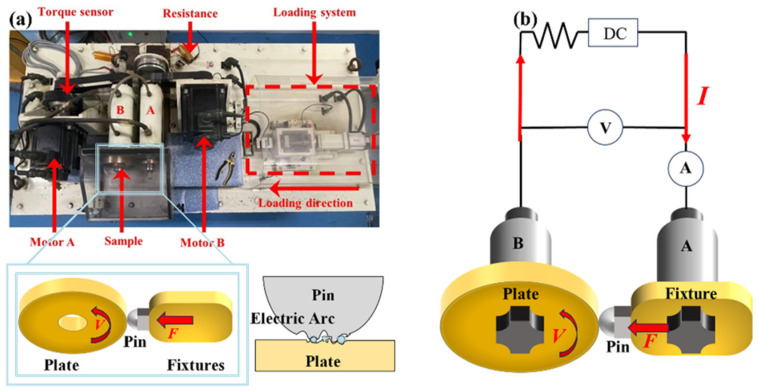
Illustrations of the test equipment. (**a**) Photograph of current-carrying friction tester, and schematic diagram; (**b**) circuit diagram of the current-carrying system.

The disk sample was fixed onto Axis-B and the pin sample was fixed onto Axis-A, as shown in Figure 2. The loading system produced vertical loading from the pin to the disk sample. This was used to simulate the preload between the rail and armature. Motor-A drove the rotation of Axis-B and the disk sample. The pin sample was able to slide on the circle of the rotated disk sample. When the AC power was loaded to the pin-on-disk tribo-pair, the test rig was capable of simulating the current-carrying sliding between the rail and armature.

**Figure 3:**
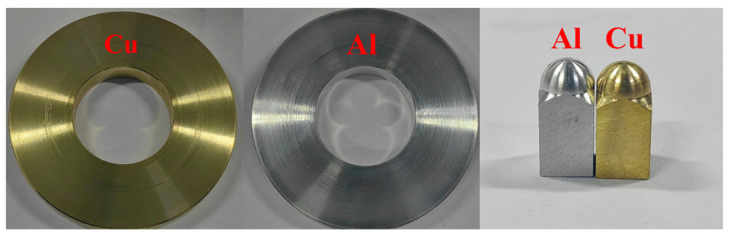
Friction-pair materials.

For the present study, we selected 7075 aluminum and H62 copper as the rail and armature materials, respectively. These materials were provided by Chinalco Luoyang Copper Processing Co., Ltd. (Luoyang, China), and then customized. They were made into plate and pin samples, as shown in Figure 3. The diameter of each plate was 80 mm. The top of each pin was processed into a hemispherical form to increase the current density during contact with the tribo-pair. The contact surfaces of the samples were polished using 800#, 1200#, and 1500# abrasive sandpaper, before the tests. After testing, the samples were cleaned in an ultrasonic cleaner for 15 min using anhydrous ethanol.

Table 1 shows the composition and content of two materials.

### 2.2. Test Method

During the armature launching process, an electric current flowed from copper to aluminum to copper. In light of this, we formed 7075-Al and H62-Cu into disks and pins to study the effect of different current directions on the friction of the materials. Five current densities were tested: 15.92 A/mm^2^, 31.84 A/mm^2^, 47.77 A/mm^2^, 58.65 A/mm^2^, and 79.61 A/mm^2^. Experimental parameters were set as follows: the normal load was 40 N, the data acquisition frequency was 50 Hz, the test time was 20 min, and the rotational speed was 300 r/min. An electronic analytical balance (FA224C, Shanghai Jingke Scientific Instrument Co., Ltd., Shanghai, China) with an accuracy of 0.1 mg was used to analyze the level of pin wear at different current densities.

During the test, the real-time friction coefficient, μ, the friction force, f, the circuit current, *I*, and the voltage, *U*, could all be measured. The contact resistance, *R*, of the current-carrying friction pair was calculated as follows:

$$R=\frac{U}{I}-R_{x}$$

where *R_x_* is the system resistance.

In this experiment, wear rates were calculated by measuring the differences between the masses of the samples before and after wear (mass-loss method).

$$Wear\;Rate=\frac{m_{1}-m_{2}}{m_{1}}100\%$$

In this formula, $m_{1}$ is the mass of the material before wear and $m_{2}$ is the mass of the material after wear.

### 2.3. Microscopic Characterization

Damage to the surface of each sample was first observed using an optical microscope (Optical Microscopy, OM, Leica DMi8C, Berlin, Germany). Then, scanning electron microscopy (SEM, Zeiss Sigma 300, Cologne, Germany) was used to observe the wear morphology of the sample’s surface. For all samples, the roughness, depth, and profile of the wear surface was observed using a white-light interference three-dimensional profilometer (White-Light Interference Three-Dimensional Profilometer, Nano Focus-μsurf expert KC-X100, Oberhausen, Germany). Content levels of oxygen, copper, aluminum, and zinc were analyzed using EDS (EDS, Smartedx, Dorsten, Germany), so that the oxidation of the wear surface and the compositional distribution could be studied.

## 3. Results and Discussion

**Figure 4:**
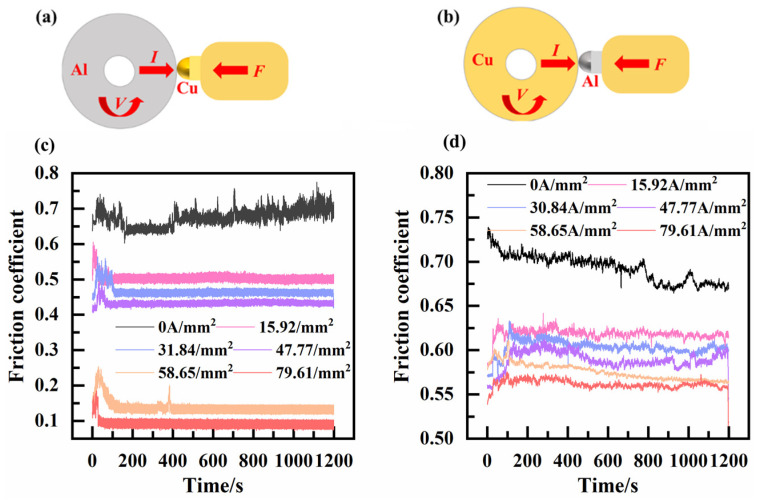
(**a**,**b**) Friction coefficients at different current densities for the (**a**) 7075-Al (anode)/H62-Cu (cathode) friction pair and (**b**) copper (anode)/7075 aluminum (cathode) friction pair. (**c**,**d**) Real-time friction coefficients for (**c**) 7075-Al (anode)/H62-Cu (cathode) and (**d**) H62-Cu (anode)/7075-Al (cathode).

Figure 4 shows the variations in coefficients of friction at different current densities. It can be observed that all the coefficients of friction decrease with increases in the current density. The coefficient of friction of the H62-Cu (anode)/7075-Al (cathode) pair fluctuates between 0.55 and 0.65. However, the coefficient of friction of the 7075-Al (anode)/H62-Cu (cathode) pair exhibits an abrupt decline to a value of 0.15.

**Figure 5:**
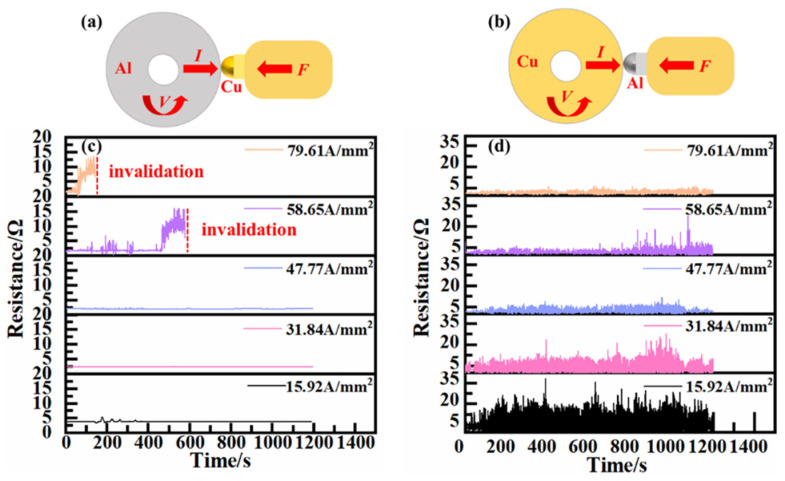
(**a**,**b**) Resistance of (**a**) 7075-Al (anode)/H62-Cu (cathode) friction pair and (**b**) copper (anode)/7075 aluminum (cathode) friction pair at different current densities. (**c**,**d**) Real-time resistance of (**c**) 7075-Al (anode)/H62-Cu (cathode) and (**d**) copper (anode)/7075 aluminum (cathode).

Figure 5 shows the resistance curves of the friction pairs under different current densities. When the current density reaches 58.65 A/mm^2^, the fluctuation in the contact resistance of the 7075-Al (anode)/H62-Cu (cathode) pair is greater, and electric conduction fails after about ten minutes. When the current density reaches 79.61 A/mm^2^, electric conduction fails after about four minutes. It can also be seen that the resistance of the H62-Cu (anode)/7075-Al (cathode) pair decreases with the increase the in current density. When the current density is 15.92 A/mm^2^, the contact resistance is about 5.91 Ω. When the current density increases to 79.61 A/mm^2^,the contact resistance is about 1.74 Ω. Overall, it can be seen from Figure 5 that, the higher the current density, the lower the fluctuation in the contact resistance [17].

**Figure 6:**
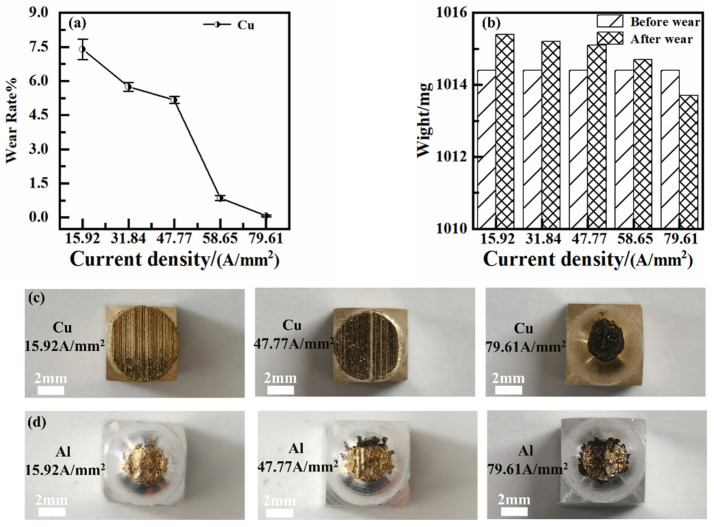
(**a**) Wear rate of copper material in the 7075-Al (anode)/H62-Cu (cathode) friction pair; (**b**) mass of aluminum material before and after wear in the H62-Cu (anode)/7075-Al (cathode) friction pair; (**c**) worn Cu pin; (**d**) worn Al pin.

Pin samples were weighed before and after testing so that the wear rates could be calculated. It was found that the wear rate of brass samples in the 7075-Al (anode)/H62-Cu (cathode) pair decreased gradually with increases in the current density, as illustrated in Figure 6 [18]. When the current density reached 58.65 A/mm^2^, the wear rate dropped abruptly. As the current density increased, the copper material adhered to the surface of the aluminum sample in the H62-Cu (anode)/7075-Al (cathode) pair, i.e., the copper material was transferred to the wear surface of the aluminum sample. This caused the mass of the aluminum sample to increase, rather than decrease. This transfer of copper material decreased gradually as the current density increased.

**Figure 7:**
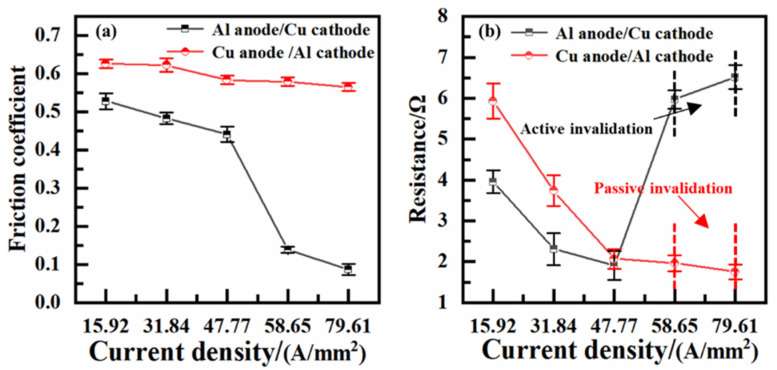
(**a**) Average friction coefficient at different current densities; (**b**) average resistance at different current densities.

As current density increased, the friction coefficients of the two pairs decreased, and the tribological performance improved, as illustrated in Figure 7. However, this improvement in tribological properties was the result of the serious electrical erosion of the materials [19]. It can also be seen in Figure 7 that, in the 7075-Al (anode)/H62-Cu (cathode) pair, the friction coefficient decreases abruptly when the current density reaches 58.65 A/mm^2^. At this time, a powerful arc was generated during friction. The powerful arc and friction heat resulted in the ablation and oxidation of the surface of the material [20]. With the increase in oxidation and the production of oxides at the contact interface, electrical invalidation between the friction pairs eventually occurred [21], and this resulted in passive invalidation on the other side. In the case of the H62-Cu (anode)/7075-Al (cathode) pair, the copper material adhered to the aluminum surface, and the damage to the rail was more serious.

### 3.1. Contact Surface Damage Mechanisms

#### 3.1.1. Surface Morphology

**Figure 8:**
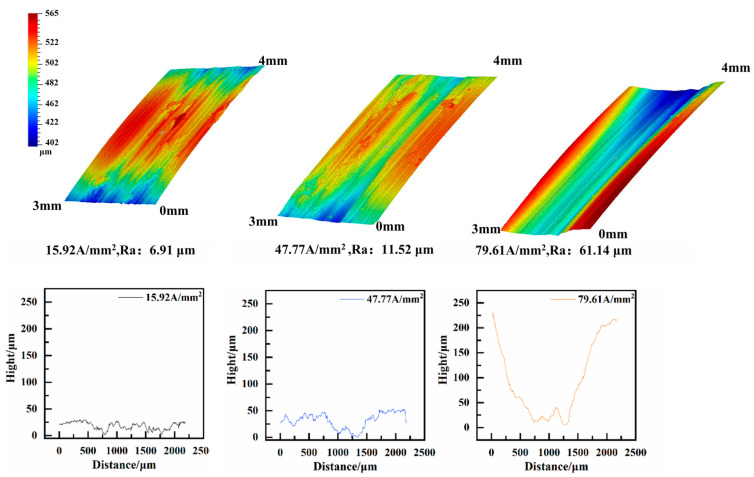

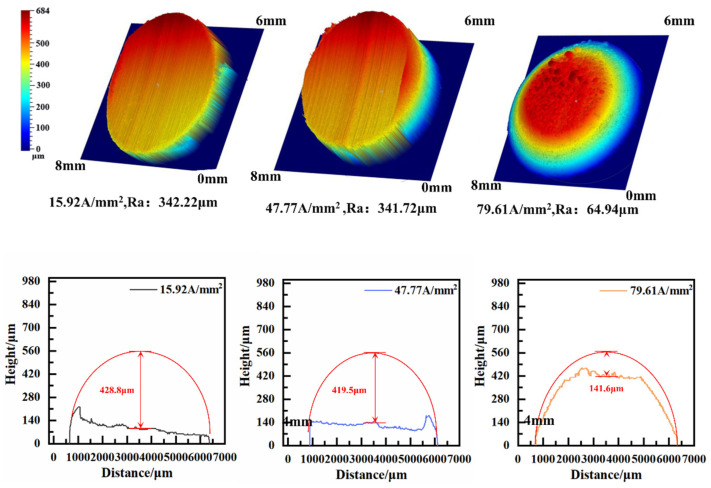
Three-dimensional morphology of the 7075-Al (anode)/H62-Cu (cathode) pair.

All measurements of roughness were taken using the middle parts of samples. As illustrated in Figure 8, the surface morphology of friction wear under different current densities was observed using a three-dimensional profilometer. The wear affecting the copper sample in the 7075-Al (anode)/H62-Cu (cathode) pair was mainly mechanical in nature when the current density was lower than 58.65 A/mm^2^. As the current density increased, the wear volume and surface roughness both decreased [22]. The wear volume and surface roughness decreased abruptly when the current density was higher than 58.65 A/mm^2^. This is because the hardness of H62-Cu is lower than that of 7075-Al, the friction wear is mainly mechanical in nature, and the arc is not obvious at such a low current density. The damage to the copper was more serious. When the current density reached 58.65 A/mm^2^, a powerful arc was produced between the contact surface of the friction pair, and the powerful arc heat caused the aluminum to melt and vaporize. Al was then oxidated and deposited on the surface of Cu [20], and this caused the wear volume to decrease.

**Figure 9:**
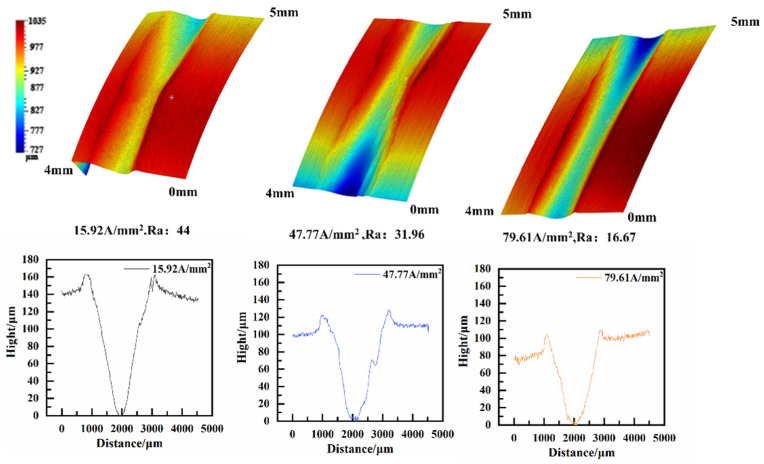

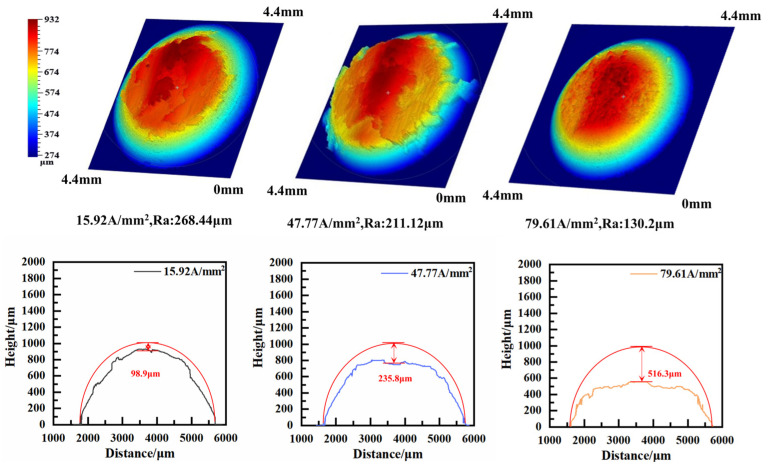
Three-dimensional morphology of the copper (anode)/7075 aluminum (cathode) pair.

In Figure 9, it can be seen that the wear volume of the aluminum specimen in the H62-Cu (anode)/7075-Al (cathode) pair increases gradually with increases in the current density. This is because the hardness of H62-Cu is lower than that of 7075-Al, the wear of copper is more serious than that of aluminum, and copper adheres to the surface of aluminum at low current densities. When the current density increases, the frequency of arcs also increases. The effects of friction heat and arc heat caused the surface temperature of the friction pair to increase; this reduces the plastic deformation resistance of the aluminum [23], resulting in the increased wear of the aluminum sample.

#### 3.1.2. Ablation Damage

**Figure 10:**
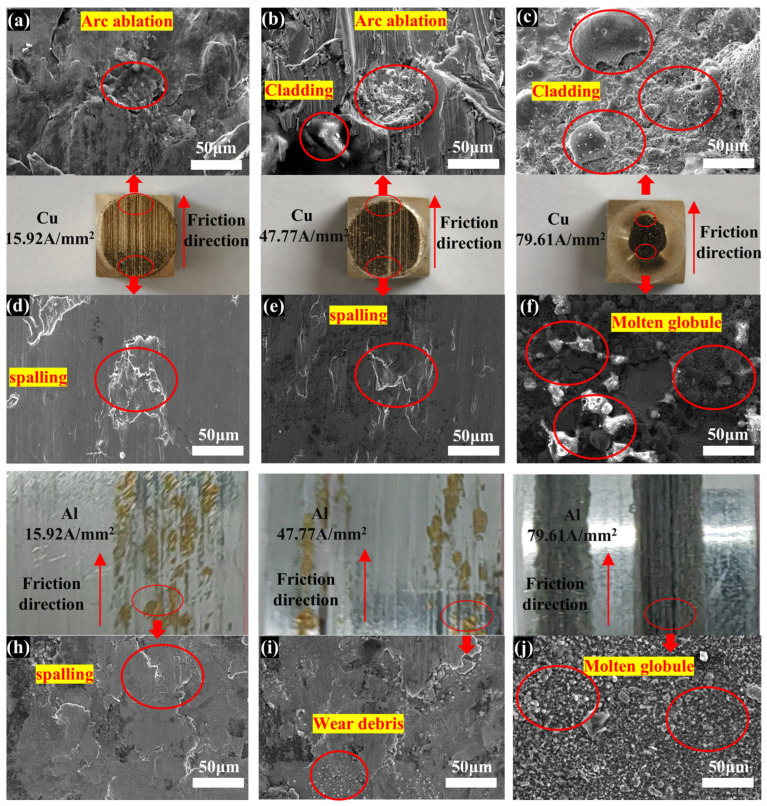
The 7075-Al (anode)/H62-Cu (cathode) electron micrographs. (**a**) The electron micrographs of the Cu at the outlet of the friction direction with a current density of 15.92 A/mm^2^. (**b**) The electron micrographs of the Cu at the outlet of the friction direction with a current density of 47.77 A/mm^2^. (**c**) The electron micrographs of the Cu at the outlet of the friction direction with a current density of 79.61 A/mm^2^. (**d**) The electron micrographs of the Cu at the inlet of the friction direction with a current density of 15.92 A/mm^2^. (**e**) The electron micrographs of the Cu at the inlet of the friction direction with a current density of 47.77 A/mm^2^. (**f**) The electron micrographs of the Cu at the inlet of the friction direction with a current density of 79.61 A/mm^2^. (**h**) The electron micrographs of the Al with a current density of 15.92 A/mm^2^. (**i**) The electron micrographs of the Al with a current density of 47.77 A/mm^2^. (**j**) The electron micrographs of the Al with a current density of 79.61 A/mm^2^.

To further study the effects of different current densities on the friction mechanism of the Cu/Al pair, the wear surfaces of samples were characterized using SEM. As illustrated in Figure 10, in the 7075-Al (anode)/H62-Cu (cathode) pair, the copper shows adhesive spalling at the initial position of the worn surface when the current density is lower than 58.65 A/mm^2^. At this time, fewer arc ablation craters can be observed at the end of the worn surface [24]. This result reflects the non-uniform character of the damage [25]. When the current density was higher than 58.65 A/mm^2^, a large area of melting appeared on the surface of the copper material. EDS analysis proved that the surface of the melting area was mainly aluminum oxide. This result shows that the powerful arc heat and friction heat cause the surface of the aluminum to melt and vaporize. The aluminum vapor was deposited and oxidized on the copper surface at this time, resulting in the formation of an aluminum deposition layer [26,27,28]. In addition, holes were found to be distributed on the surface of the deposition layer. The formation of these holes was due to the hydrolysis reaction of aluminum liquid with atmospheric water vapor. As cooling aluminum melt was deposited, the water vapor formed holes in the deposited layer [27,29,30].

**Figure 11:**
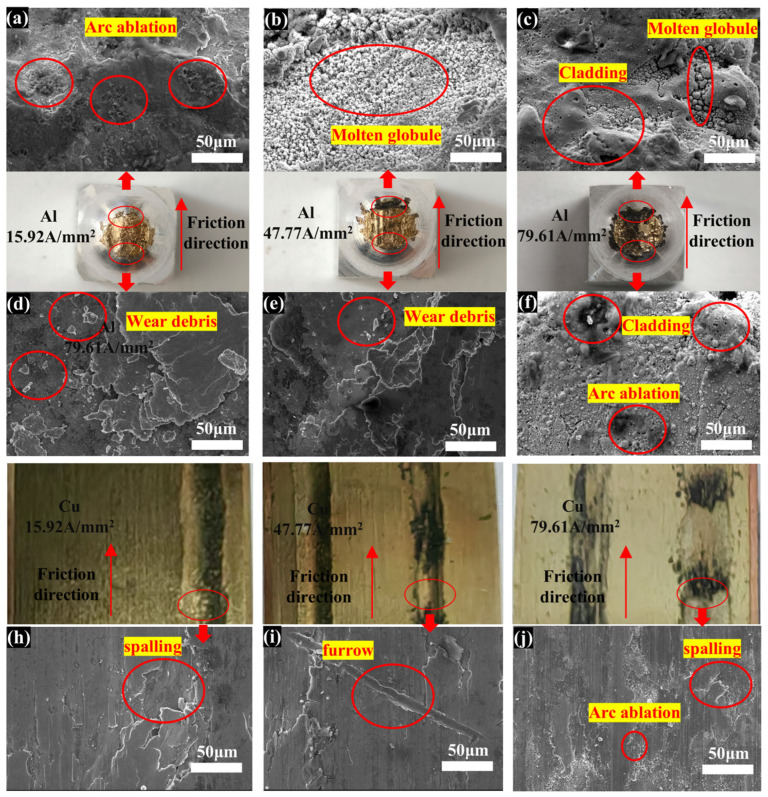
H62-Cu (anode)/7075-Al (cathode) electron microscope diagrams. (**a**) The electron micrographs of the Al at the outlet of the friction direction with a current density of 15.92 A/mm^2^. (**b**) The electron micrographs of the Al at the outlet of the friction direction with a current density of 47.77 A/mm^2^. (**c**) The electron micrographs of the Al at the outlet of the friction direction with a current density of 79.61 A/mm^2^. (**d**) The electron micrographs of the Al at the inlet of the friction direction with a current density of 15.92 A/mm^2^. (**e**) The electron micrographs of the Al at the inlet of the friction direction with a current density of 47.77 A/mm^2^. (**f**) The electron micrographs of the Al at the inlet of the friction direction with a current density of 79.61 A/mm^2^. (**h**) The electron micrographs of the Cu with a current density of 15.92 A/mm^2^. (**i**) The electron micrographs of the Cu with a current density of 47.77 A/mm^2^. (**j**) The electron micrographs of the Cu with a current density of 79.61 A/mm^2^.

At a low current density, the aluminum in the H62-Cu (anode)/7075-Al (cathode) pair exhibited scale-like damage at the initial position of the worn surface (Figure 11). In a sliding friction process, one part of the damage is scale-like, resulting from the shedding of abrasive debris; the other part of the damage involves the accumulation of a large block on the surface of the specimen [31]. In addition, a wide area of arc ablation was exhibited at the end of the worn surface. As the current density increased, arc ablation occurred over the entire area of the worn surface, with the most serious ablation being exhibited at the end of the worn surface [32].

#### 3.1.3. Surface Oxidation

**Figure 12:**
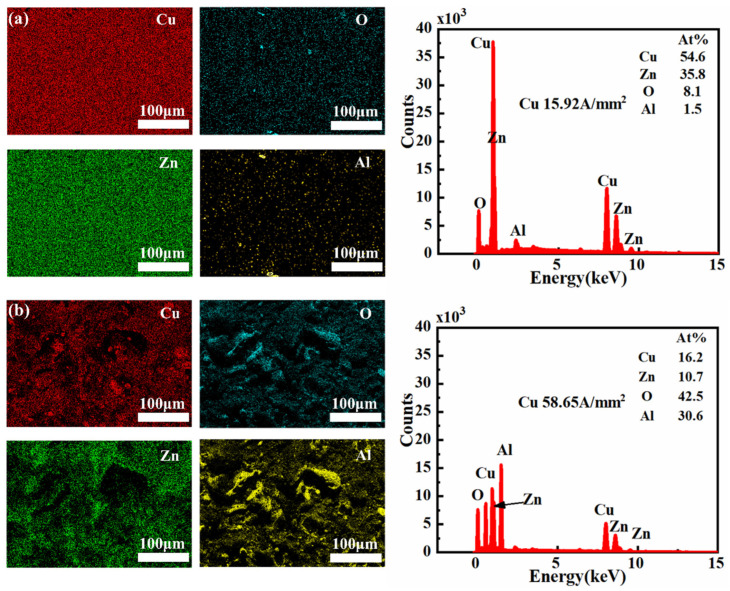
The 7075-Al (anode)/H62-Cu (cathode) EDS images and analyses. (**a**) EDS image of Cu at 15.92 A/mm^2^ current density. (**b**) EDS image of Cu at 58.65 A/mm^2^ current density.

The composition of wear surfaces at different current densities was analyzed using EDS. At a current density of 15.92 A/mm^2^, the wear surface of the 7075-Al (anode)/H62-Cu (cathode) pair was found to be composed of 54.6% Cu, 8.1% oxygen, and 1.5% aluminum. However, when the current density was increased to 58.65 A/mm^2^, the copper content on the wear surface decreased to 16.2%, while the proportions of oxygen and aluminum both increased significantly, to 42.5% and 30.6%, respectively (Figure 12). This shows that the powerful arc heat caused the aluminum to be melted and vaporized; it was then deposited on the copper surface, promoting oxidation [32]. At low current densities, due to severe mechanical wear, only small amounts of aluminum adhered to the surface of the copper pin.

**Figure 13:**
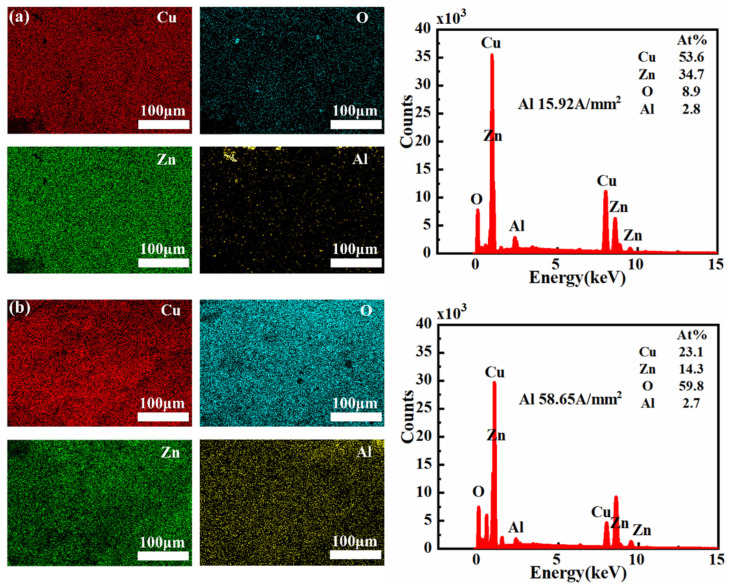
H62-Cu (anode)/7075-Al (cathode) EDS images and analyses. (**a**) EDS image of Al at 15.92 A/mm^2^ current density. (**b**) EDS image of Al at 58.65 A/mm^2^ current density.

Aluminum in the H62-Cu (anode)/7075-Al (cathode) pair had 53.6% copper, 8.9% oxygen, and 2.8% aluminum on the wear surface at a current density of 15.92 A/mm^2^. The surface of the aluminum sample was therefore covered by copper, making it difficult to observe the aluminum on the surface. However, when the current density increased to 58.65 A/mm^2^, the copper content on the wear surface decreased to 23.1%, while the oxygen content increased to 59.8%. This shows that the high current density reduced the transfer of copper and promoted the oxidation of the material. Although the image of the aluminum appears quite bright, as illustrated in Figure 13 its content level is only 2.7%. This is due to the higher proportion of other elements on the surface of the aluminum pin, including copper, which is also very bright in the image, but accounts for 23.1% of the total content. The images of the other elements are all brighter than that of aluminum. The proportion of oxygen alone was 59.8%. This explains the relatively low numerical value for the aluminum content.

Failure: The application of an electric current to a metallic material increases the plastic deformability of the material due to the electrophysical effect. (The deformation resistance of metal materials decreases and the plasticity increases under the action of an electric current) [33]. Many studies have shown that electro-plasticity in metals is not caused by a single thermal or non-thermal effect. Rather, it is caused by both thermal and various non-thermal effects [34,35,36]. At the same time, the phenomenon of electrical migration induced by electric currents cannot be ignored, as this is known to cause material damage and corresponding function failure. As electrons move from the cathode to the anode, they collide with metal ions and transfer momentum to individual atoms, moving them in the direction of the electron wind [37]. In addition, the direction of the current influences both the effect of electromigration between the materials and the differing sensitivities of material microstructures to electro-plastic effects [38]. This is also the reason for the differences between the two different pairs tested in the present study, with respect to both experimental phenomena and the results obtained during the current-carrying friction test. In the case of the 7075-Al (anode)/H62-Cu (cathode) pair, electromigration was not significant at low current densities, and the electro-plastic effect caused the plastic deformation of copper to increase [39]. The plastic deformation of copper is further enhanced by the effects of resistance heat and arc heat [40,41,42]. The increased temperature of the copper surface promotes oxidative wear [43] and fatigue wear [44,45], causing the surface of the copper pin to show scale-like damage. The friable oxide layer on the surface is prone to cracking and breaking into abrasive particles when subjected to ongoing frictional stress. The increased temperature impairs the mechanical and physical properties of the surrounding materials, making them more susceptible to fracture and fatigue delamination. Copper is constantly peeled off, and its wear area becomes larger, causing the area of contact between copper and aluminum materials to increase. Electronic movement is smoother and electrical conductivity is enhanced between copper and aluminum materials. This results in less arcs between the copper/aluminum friction pair, lower contact resistance, and insignificant resistance fluctuations. When the current density is increased, aluminum is easily affected by electromigration; it then transfers to copper, causing defects [37]. At the same time, because the conductivity of aluminum is poor, compared with copper, the electromigration of electrons from copper to aluminum is blocked. At this moment, a large arc is generated with powerful arc heat. The effects of arc heat and Joule heat, combined with the low melting point of aluminum, result in the aluminum being vaporized by ablation and oxidized along the friction direction on the surface of the friction sub-surface. The friction interface forms an oxidation film, which causes the friction coefficient to decrease dramatically and the resistance fluctuation to increase sharply. In the H62-Cu (anode)/7075-Al (cathode) mating pair, electromigration is not significant at a low current density; the electric current flows from the aluminum to the copper, and copper has better electrical conductivity than aluminum. During the electromigration process, electrons converge in the aluminum material and are released in the copper material. At the same time, the electro-plastic effect enhances the plastic deformation ability of copper; this material is constantly peeled off under the action of shear force and adheres to the surface of the aluminum material, resulting in significantly improved electromigration between the two materials. When the current density increases, copper is more mobile than aluminum under the influence of electromigration [46]. The effects of electrophysical plasticity then cause the transfer of copper to aluminum to increase. Under subjection to arc heat and friction heat, oxidization increases.

**Figure 14:**
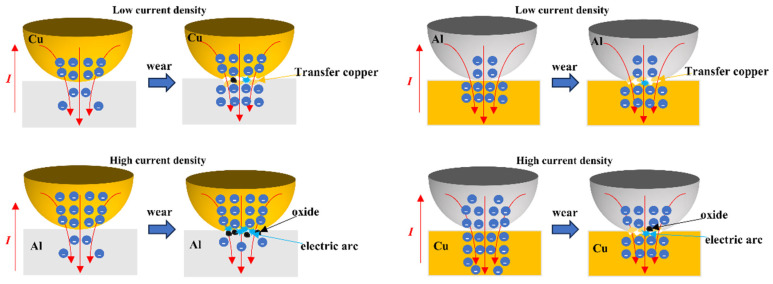
Material Damage Model.

As shown in Figure 14, we present the damage models of these two mating pairs under low and high current densities.

**Figure 15:**
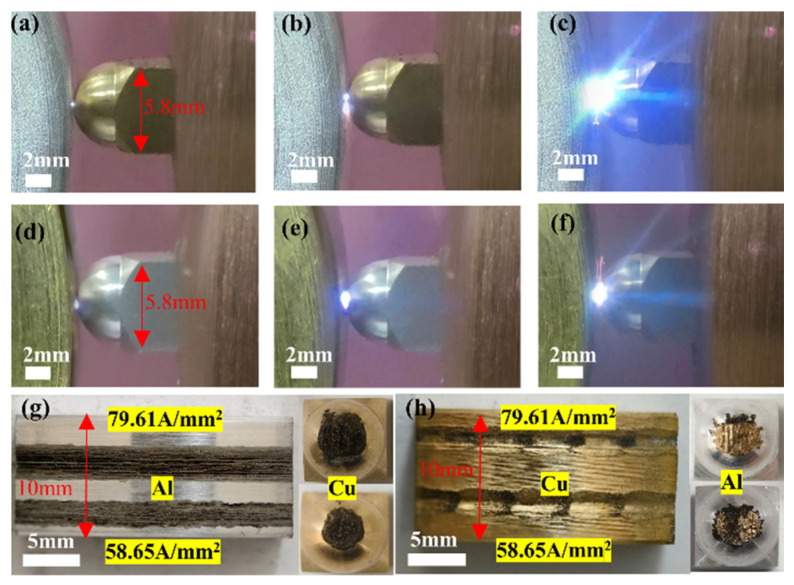
(**a**–**c**) Arc diagrams of the 7075-Al (anode)/H62-Cu (cathode) pair at (**a**) 15.92 A/mm^2^; (**b**) 47.77 A/mm^2^; and (**c**) 79.61 A/mm^2^ current densities. (**d**–**f**) Arc diagrams of the H62-Cu (anode)/707-Al (cathode) pair at (**d**) 15.92 A/mm^2^; (**e**) 47.77 A/mm^2^; and (**f**) 79.61 A/mm^2^ current densities. (**g**) The 7075-Al (anode)/H62-Cu (cathode) friction pair at 58.65 A/mm^2^ and 79.61 A/mm^2^. (**h**) The H62-Cu (anode)/7075-Al (cathode) friction pair at 58.65 A/mm^2^ and 79.61 A/mm^2^.

Figure 15 shows the size and morphology of the electric arc, as well as the damage characteristics of the material, under different current densities.

This experiment was conducted in a non-EMI environment, and there are certain differences between our results and those of related studies carried out by other scholars in the past. Dinesh G et al. [14] conducted tests using a linear tribo-simulator. Because of its low sliding speed (0.15 m/s), the impact of arc generation was not observed in their experiment. In addition, there is a lack of research on the damage to materials caused by polarity changes. Jyh-Chia and Vook [13] tested aluminum wire brushes on rotating copper slip rings and copper wire brushes on aluminum slip rings in a high vacuum environment. Such a condition does not match the actual application environment in which electromagnetic launch technology is used, i.e., an atmospheric environment.

## 4. Conclusions

In this paper, we studied the effects of current density and direction on the tribological properties of H62-Cu/7075-Al. The surface morphology, roughness, and composition of the wear surface were all analyzed. The direction of the current was found to affect electromigration between the two tracks, resulting in differences in the damage caused to the armature rail interface. Our conclusions can be summarized as follows:

(1) When the current flowed from 7075-Al to H62-Cu, the effects of current density were different. Shear force resulted in serious mechanical damage at a low current density. Arc erosion was produced at a high current density. Under subjection to strong arc heat and friction heat, 7075-Al melted and vaporized. It was then oxidated and deposited on the surface of H62-Cu. The friction coefficient was therefore reduced, promoting the lubrication of the oxides. At the same time, there was a deterioration in electrical conductivity, because of the poor conductivity of the oxides.
(2) When the current flowed from H62-Cu to 7075-Al, the electrical transport property was much improved, compared with when the current flowed from 7075-Al to H62-Cu. Under the influence of electro-plasticity, the plastic deformation ability of H62-Cu was enhanced. It peeled off and adhered to the surface of the 7075-Al. Cu and its oxides adhered even more heavily to the 7075-Al sample.
(3) In the 7075-Al (cathode)/H62-Cu (anode) pair, the material surface was severely eroded due to the large arc. A dense oxide film formed on the material contact surface, leading to the failure of electrical conduction between the materials. In the H62-Cu (anode)/7075-Al (cathode) pair, the copper material suffered severe mechanical damage. The stability of contact between materials decreased, resulting in Hertzian contact failure.
(4) Non-uniform damage was detected on the worn surfaces of the tribo-pairs along the sliding direction. Forms of mechanical damage, such as spalling, wear debris, and furrows, were always observed at the initial positions of the worn surfaces. On the other hand, arc erosion was always produced at the ends of the worn surfaces.

From the results of this experiment, it can be seen that polarity has an impact on the unevenness of material damage. On the one hand, the failure of electrical conduction caused by arc erosion damage reduces the launch speed of the armature. On the other hand, severe mechanical damage to the track affects the secondary firing of the EMRG. To solve this problem, the material design can be changed. For example, replacing one side of the track with a copper carbon steel gradient material can increase electrochemical impedance without affecting electrical conductivity, thereby improving corrosion resistance [47]. Even after the surface copper layer is destroyed, the gradient material can protect the matrix and improve the service life of the material [48,49]. Additionally, copper-based composite materials may be used for the other side of the track. Materials such as Cu-Cr [50], Cu-W [51] and Cu/Al_2_O_3_ [52] all have high strength, in addition to good arc ablation resistance, electrical conductivity, and friction resistance [53]. The use of such materials will greatly reduce rail damage, reduce arc generation, and improve launch efficiency. Further research into copper-based composite materials may play an important role in the future development of EMRGs.

## 5. Prospects

In recent years, researchers from different countries have made great efforts in the study of EMRGs. Great progress has been made in terms of the materials, structural design and simulation analysis. The failure of EMRGs is still one of the most urgent problems to be solved. The key to solving related problems is to identify the causes of failure and study damage mechanisms. Differences in damage to the armature on the two sides of the track cause armature deviation during the process of launch, affecting firing accuracy and decreasing launch safety. In light of this, further studies of the influence of current direction on materials should be carried out. A scholarly understanding of the mechanisms involved in current-carrying tribology under severe working conditions should also be encouraged, so that EMRG technology may advance from laboratory testing to real-world engineering applications.

## Figures and Tables

**Table 1:** Composition of friction pairs.

	**Si**	**Fe**	**Cu**	**Mn**	**Mg**	**Cr**	**Zn**	**Zr**	**Ti**
7075Al	0.40	0.50	1.2–2.0	0.30	2.1–2.9	0.18–0.28	5.1–6.1	0.05	0.20
	**Cu**	**Fe**	**Pb**	**Si**	**Ni**	**B**	**As**	**Zn**	
H62	63.5	0.15	0.08	-	-	-	-	tolerance	

## Data Availability

The original contributions presented in the study are included in the article, further inquiries can be directed to the corresponding author.
